# Bidirectional ventricular tachycardia due to digoxin-diuretic interaction in post-cardiac surgery patient: a case report

**DOI:** 10.47487/apcyccv.v5i2.362

**Published:** 2024-06-24

**Authors:** José Martín Alanís-Naranjo, Kevin David Aragón-Ontiveros, Julio César Rivera-Hermosillo, Virginia Campos-Garcilazo

**Affiliations:** 1 Hospital Regional Primero de Octubre ISSSTE, Mexico City, Mexico. Hospital Regional Primero de Octubre ISSSTE Mexico City Mexico

**Keywords:** Tachycardia, Ventricular, Digoxin, Diuretics, Arrhythmias, Cardiac, Thoracic Surgery, Taquicardia Ventricular, Digoxina, Diuréticos, Arritmias Cardíacas, Cirugía Cardiaca

## Abstract

Bidirectional ventricular tachycardia (BVT) is a rare form of malignant ventricular arrhythmia characterized by beat-to-beat alternation in the QRS axis. BVT is a hallmark of digitalis toxicity, but digoxin-induced BVT secondary to digoxin-diuretic interaction in cardiac surgery patients is not widely reported. We present the case of a 62-year-old woman undergoing mitral valve replacement with tricuspid annuloplasty who developed postoperative congestive heart failure and vasoplegic syndrome requiring norepinephrine, vasopressin, and loop diuretics. During postoperative care, she presented atrial fibrillation with rapid ventricular response, achieving rate control with digoxin, but later displayed hemodynamically stable BVT associated with digitalis toxicity. The case highlights the importance of physicians monitoring digoxin toxicity when prescribing digoxin to patients with a diuretic regimen, particularly loop diuretics. During digoxin-induced-BVT, supportive treatment, including discontinuing digitalis coupled with potassium and magnesium supplements, can be considered as long as digoxin-specific antibodies are unavailable, and the patient is hemodynamically stable.

## RESUMEN

La taquicardia ventricular bidireccional (TVB) es una arritmia rara caracterizada por alternancia latido a latido en el eje QRS. La TVB es característica de intoxicación digitálica; sin embargo, la TVB secundaria a interacción digoxina-diurético en pacientes posoperados de cirugía cardíaca no se ha reportado ampliamente. Presentamos el caso de una mujer de 62 años sometida a cirugía cardiaca que desarrolló falla cardiaca congestiva y síndrome vasopléjico en el posoperatorio por lo que requirió noradrenalina, vasopresina y diurético de asa. Durante la hospitalización presentó fibrilación auricular con respuesta ventricular rápida; se logró control con digoxina, pero posteriormente presentó TVB asociada a intoxicación digitálica. Este caso resalta la importancia de detectar intoxicación digitálica durante la prescripción de digoxina a pacientes con un régimen diurético, especialmente diuréticos de asa. Durante la TVB inducida por digoxina, el tratamiento de soporte se puede considerar cuando no haya disponible anticuerpos específicos para digoxina y el paciente este hemodinámicamente estable.

## Introduction

Bidirectional ventricular tachycardia (BVT) is a rare form of malignant ventricular arrhythmia characterized by beat-to-beat alternation in the QRS axis ^1^. BVT can be caused by various factors, including digitalis toxicity, aconitine toxicity, Andersen- Tawil syndrome, hypokalemic periodic paralysis, myocardial infarction, myocarditis, left ventricular hypertrophy, and pheochromocytoma ^2^. Digoxin-diuretic interaction has been reported to cause digoxin intoxication with serious cardiac arrhythmias, particularly loop diuretics ^3^. BVT is a hallmark of digitalis toxicity, but digoxin-induced BVT secondary to digoxin-diuretic interaction in cardiac surgery patients is not widely reported ^4^.

**Figure 1:**
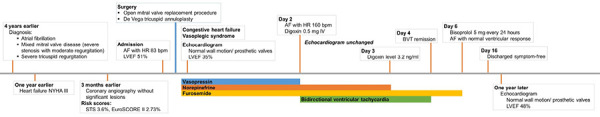
Clinical presentation and evolution of digoxin-induced bidirectional ventricular tachycardia secondary to digoxin-diuretic interaction in a cardiac surgery patient. AF: atrial fibrillation, LVEF: left ventricular ejection fraction, HR: heart rate, BVT: bidirectional ventricular tachycardia.

We present the case of a 62-year-old woman undergoing mitral valve replacement with tricuspid annuloplasty who developed postoperative congestive heart failure and vasoplegic syndrome requiring norepinephrine, vasopressin, and loop diuretics. During postoperative care, she presented atrial fibrillation with rapid ventricular response, achieving rate control with digoxin, but later displayed hemodynamically stable BVT associated with digitalis toxicity. Figure 1 depicts the clinical presentation and evolution of the case.

## Case report

**Figure 2:**
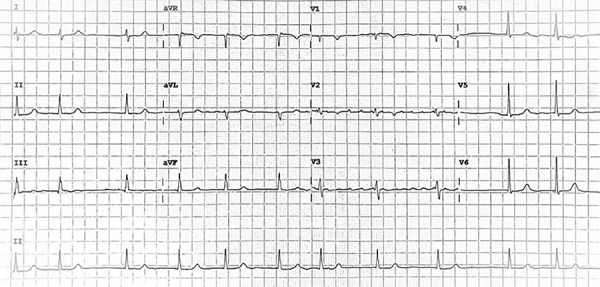
ECG on admission: Atrial fibrillation with Normal ventricular response.

A 62-year-old woman with a medical history of permanent atrial fibrillation, heart failure NYHA III, mixed mitral valve disease (severe stenosis with moderate regurgitation), and severe tricuspid regurgitation was admitted for scheduled valve surgery. The coronary angiography performed three months earlier showed no significant lesions. The surgical risk scores revealed a low-risk scenario: STS 3.6% and EuroSCORE II 2.73%. Her medication regimen included metoprolol tartrate 50 mg every 12 hours to control atrial fibrillation rate and furosemide 40 mg every 12 hours. At admission, the electrocardiogram (ECG) showed atrial fibrillation with normal ventricular response (Heart rate [HR] 83 bpm, (Figure 2) with an echocardiogram with a left ventricular ejection fraction (LVEF) of 51%.

The following day, the patient underwent an open mitral valve replacement procedure and a De Vega tricuspid annuloplasty. The transoperative period was free of malignant arrhythmias, the aortic cross-clamp lasted 80 minutes, the aortic impingement lasted 56 minutes, and the cardioplegic arrest was performed using Custodiol ® 1000 ml. She was admitted to the coronary care unit and developed congestive heart failure and vasoplegic syndrome, requiring norepinephrine, vasopressin, and furosemide 60 mg IV every 6 hours. The postoperative blood test revealed an N-terminal pro-brain natriuretic peptide level of 10647 pg/ml and a troponin level of 4400 ng/L. The control electrocardiogram’s T wave and ST segment did not change compared to the initial ECG. The echocardiogram had no abnormalities in wall motion, but the LVEF dropped to 35%; mitral prosthetic valve showed normal function: peak velocity (Vmax) of 1.86 m/s, mean pressure gradient 4 mmHg, effective orifice area (EOA) of 2.9 cm^2^, index EOA 1.54 cm^2^/m^2^.

**Figure 3:**
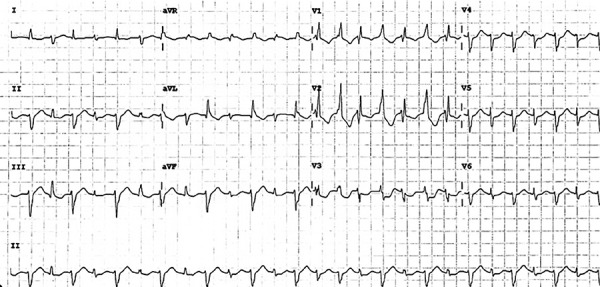
ECG with bidirectional ventricular tachycardia: Narrower QRS with right axis deviation alternating on a beat-to-beat with a wider QRS with left axis deviation.

On postoperative day 2, the patient continued the diuretic regimen with norepinephrine and developed atrial fibrillation with rapid ventricular response (HR 160 bpm). Digoxin was used with a 0.5 mg IV dose achieving rate control. After four hours, the patient developed BVT without hemodynamic instability (Figure 3). The laboratory results revealed a normal thyroid function, no electrolyte disturbances (potassium level of 4.2 mEq/L), a normal creatinine level (0.3 mg/dl), and a drop in Troponin level (1560 ng/L). A new echocardiogram showed no differences compared to the initial postoperative echocardiogram.

At 24 hours following the arrhythmia (postoperative day 3), the digoxin level was 3.2 ng/ml. Digoxin-specific antibody was unavailable in our unit, so we decided to continue with supportive care, discontinuing digoxin, prescribing potassium/magnesium supplements, maintaining urine output >1.5 ml/kg/hour, and monitoring electrolyte imbalances. The vasopressor was retired the same day, maintaining mean arterial pressure >70 mmHg and an average HR of 80 to 90 bpm.

**Figure 4:**
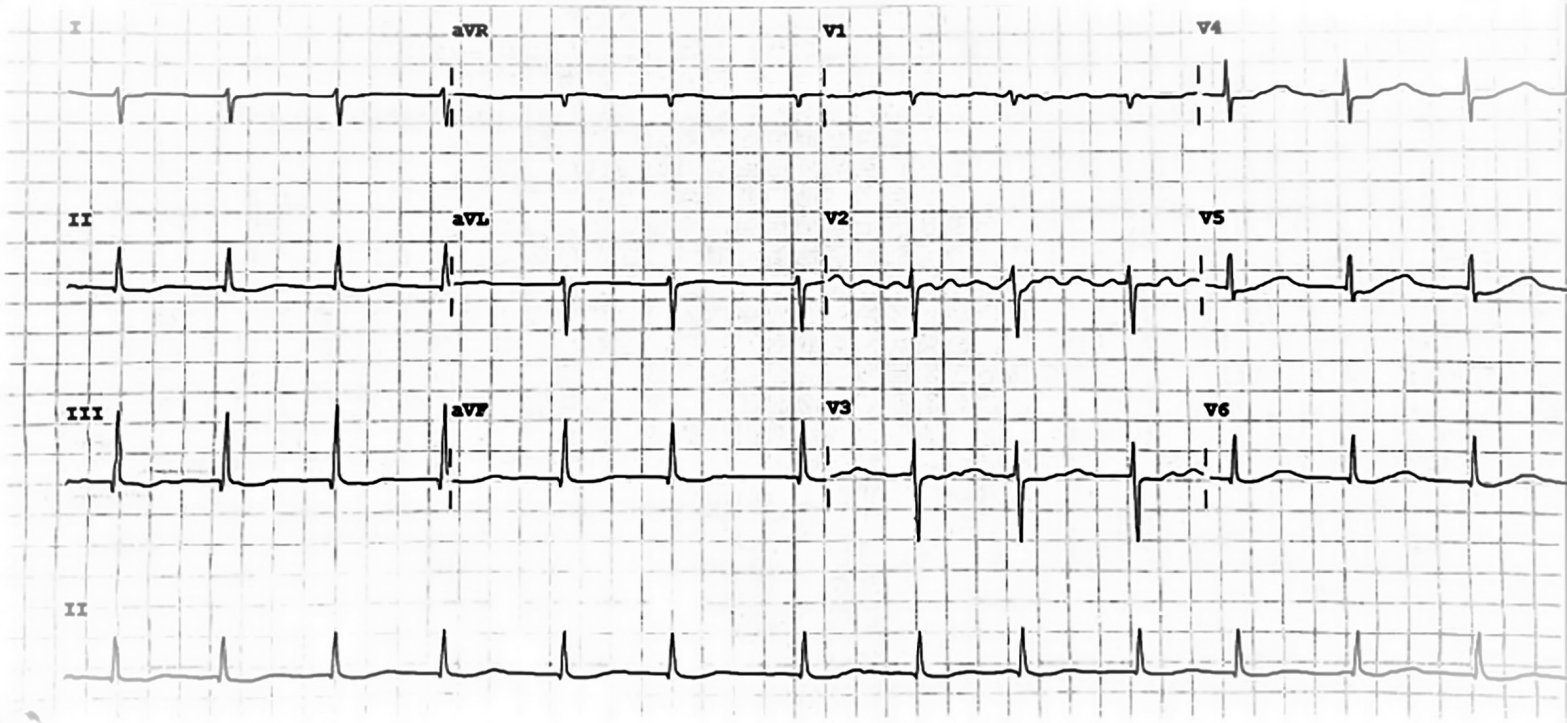
ECG at discharge: Atrial fibrillation with Normal ventricular response.

The arrhythmia resolved in the following 48 hours of the initial episode of BVT (postoperative day 4); during this period, five episodes of hemodynamically stable BVT were observed, lasting on average two minutes each, and blood tests were within normal range. Upon remission of fluid overload on postoperative day 6, diuretic therapy was discontinued, and oral beta-blocker (Bisoprolol 5 mg every 24 hours) was initiated. Atrial fibrillation continued with normal ventricular response during the rest hospital stay (Figure 4). She was discharged symptom-free on postoperative day 18 for further cardiac rehabilitation. A one-year follow-up transthoracic echocardiogram continued to report normal prosthetic valve function, no abnormalities of wall motion, and an improvement in LVEF of 48%.

## Discussion

Described by Schwensen in 1922 in a patient suffering from digitalis toxicity, BVT is a rare and severe form of ventricular tachycardia that exhibits typical ECG manifestations similar to our case: 1) two QRS morphologies alternate beat-to-beat in the same limb lead, 2) chest leads often display alternate left and right bundle branch block-like morphologies, 3) ventricular rate is 140 to 180 bpm, R-R interval is regular or alternate in length, 4) attacks are usually non-permanent or transient and last only seconds to minutes, can terminate spontaneously and be recurrent, and 5) lead V1 exhibit QS or R morphology ^2^.

Several hypotheses have been proposed to explain the mechanism of BVT. There is the hypothesis that ping-pong physiology is the predominant mechanism of BVT with two or more ventricular foci with varying trigger thresholds. Stress leads to increased sinus rhythm, during which intracellular calcium overload delays after depolarization. When the threshold is reached, one ventricular site is activated first, followed by a second one that reciprocally activates the first. This can lead to BVT, in which alternating morphologies can differ by width, axis, or bundle-branch block-like morphology according to the locations of these foci ^2^^,^^5^^,^^6^.

In the literature is known that digoxin-diuretic interactions may contribute to cardiac arrhythmias but exist few studies demonstrating a clear cause-effect relationship ^3^. Digoxin reversibly inhibits sodium-potassium ATPase (Na, K-ATPase, or the Na-K pump), thus inhibiting sodium from being pumped out of cells and potassium from being pumped in. In addition, potassium competes with digoxin for binding to the Na-K pump. Therefore, as diuretics reduce the serum potassium concentration, digoxin inhibition of the Na-K pump is further facilitated. Consequently, depletion of intracellular potassium might occur, associated with digoxin-induced arrhythmia ^3^^,^^7^. According to Wang et al., digoxin intoxication is significantly increased by more than threefold when combined with any diuretic. When analyzing the diuretics prescribed, loop diuretics had the most significant risk (OR 2.97, 95% CI 2.35,3.75) ^3^.

Despite limited information concerning digoxin toxicity following cardiac surgery, it is suggested that the etiology of arrhythmia may be the result of increased sensitivity to digitalis glycosides or the effects of surgical trauma, although the exact mechanism has not been determined. Digoxin-induced arrhythmias are difficult to distinguish with certainty from spontaneous arrhythmias in most postoperative patients ^8^.

The treatment of BVT should be based on the etiology and implemented as soon as possible ^2^^,^^9^. In the case of BVT caused by digitalis toxicity, digitalis should be discontinued immediately, coupled with potassium and magnesium supplements. In a severe digoxin overdose, antigen-binding fragments are the preferred treatment. Digitalis poisoning often causes tachyarrhythmia and slow arrhythmias, making amiodarone less desirable. The use of anti-arrhythmic drugs, such as lidocaine and amiodarone, during the active treatment of the primary disease, applies to BVT caused by coronary heart disease and cardiomyopathy. In a tachycardia attack, pacing therapy is an effective method of terminating it, and electric cardioversion is not appropriate ^2^. Phenytoin has been reported to be an effective and readily available treatment for BVT, with no recurrence of arrhythmia after treatment. The anti-arrhythmic inhibits digitalis binding to the sodium-potassium-ATPase pump and antagonizes digitalis-induced depolarization delays ^10^.

In this case, a usual digoxin dose for rate control of atrial fibrillation (0.5 mg) was administered to a post-cardiac surgery patient who suffered from vasoplegic syndrome, showed a drop on LVEF in echocardiogram and was on a diuretic regimen due to congestive heart failure. Besides electrolyte abnormalities being absent, no other typical factors that favor the development of BVT were identified (thyroid disease, renal failure, hypercalcemia, alkalosis, hypoxemia, acidosis); therefore, digoxin-diuretic interaction was considered a likely cause of digoxin-induced BVT. Based on the mechanisms previously discussed, we assumed that increased sensitivity to digitalis glycosides or surgical trauma associated with cardiac surgery coupled with ongoing diuretic therapy potentiated digoxin toxicity.

In conclusion, the clinician should monitor digoxin toxicity when prescribing digoxin to patients with a diuretic regimen, especially loop diuretics. During digoxin-induced-BVT, supportive treatment, including discontinuation of digitalis coupled with potassium and magnesium supplements, can be considered as long as digoxin-specific antibodies are unavailable, and the patient is hemodynamically stable.
